# Ultrasound-guided percutaneous microwave ablation for adenomyosis: efficacy of treatment and effect on ovarian function

**DOI:** 10.1038/srep10034

**Published:** 2015-05-05

**Authors:** Yang Yu, Zhang Jing, Han Zhi-yu, Ma Xia, Hao Yan-li, Xu Chang-tao, Xu Rui-fang, Zhang Bing-song

**Affiliations:** 1 Department of Interventional Ultrasound, Chinese PLA General Hospital; 2 Department of Ultrasound, Beijing Friendship Hospital, Capital Medical University. (This study was performed at Chinese PLA General Hospital).

## Abstract

A total of 142 premenopausal women with symptomatic adenomyosis underwent ultrasound (US)-guided percutaneous microwave ablation (PMWA) at the Chinese PLA General Hospital. This study aimed to evaluate changes in serum pituitary, gonadal hormone and cancer antigen 125 (CA125) levels after US-guided PMWA. Therefore, estradiol (E_2_), follicle-stimulating hormone (FSH), prolactin (PRL) and CA125 levels were evaluated before ablation and at 3, 6, 9 and 12 months after ablation. No significant differences were observed in the E_2_ and FSH levels pre-ablation and during follow-up (E_2_: *p* = 0.933, *p* = 0.987, *p* = 0.106, *p* = 0.936; FSH: *p* = 0.552, *p* = 0.295, *p* = 0.414, *p* = 0.760). The mean absolute values of serum CA125 and PRL were significantly decreased at 3, 6, 9 and 12 months after ablation (CA125: *p* < 0.001, *p* < 0.001, *p* < 0.001, *p* = 0.003; PRL: *p* < 0.001, *p* < 0.001, *p* < 0.001, *p* < 0.001). A significant correlation between changes in CA125 levels and uterine volume was found (*p* < 0.001). No evidence of a decline in ovarian function was observed after US-guided PMWA.

Adenomyosis is a common disease among women of childbearing age. Menorrhagia and dysmenorrhea are two of the most common symptoms and always require clinical intervention. Suppressive hormonal treatments can temporarily relieve symptoms, but these treatments interfere with patient fertility^1^,^2^. Hysterectomy is the standard treatment for adenomyosis^3^ but may not be accepted by every patient of reproductive age.

Conservative treatments for adenomyosis are rapidly being developed. Ensuring the efficiency of treatment without negatively affecting ovarian function remains a longstanding challenge in these patients^4^. Ultrasound (US)-guided percutaneous microwave ablation (PMWA) has been used in the treatment of symptomatic adenomyosis and fibroids^5^,^6^,^7^,^8^,^9^, which has produced satisfactory clinical outcomes. However, changes in serum hormone levels after PMWA have not been thoroughly studied. In previous studies, follicle-stimulating hormone (FSH) and estradiol (E_2_) levels were typically assessed to determine the remaining ovarian reserve^10^. In addition, clinical signs of the menopausal transition, such as amenorrhea, hot flashes, mood disorders, and vaginal dryness, may also be used to assess ovarian function^11^. On the other hand, the measurement of serum cancer antigen 125 (CA125) and PRL levels are useful in monitoring adenomyosis and the effects of treatment^12^,^13^,^14^; therefore, changes in this hormone may be used to evaluate the efficacy of treatments.

Thus, this study was undertaken to analyze the efficacy of treatment and effect on ovarian function of PMWA for adenomyosis.

## Materials and Methods

### Patients

From February 2011 to May 2014, a total of 278 premenopausal women with symptomatic adenomyosis underwent US-guided PMWA at the Department of Interventional Ultrasonics, General Hospital of Chinese PLA, Beijing, China. All of the patients were diagnosed with adenomyosis based on ultrasound and magnetic resonance imaging (MRI). The criteria for this study of adenomyosis on ultrasound were: (1) No distinction of the endometrial-myometrial junction; (2) asymmetry of the anterior and posterior myometrium; (3) subendometrial myometrial striations; (4) myometrial cysts; and (5) heterogeneous myometrial echotexture^15^. The criteria for this study of adenomyosis on MRI were: (1) a myometrial mass with indistinct margins of primarily low intensity, (2) diffuse or local widening of junctional zones on T2 weighted images, (3) junctional zone wider than 35 mm, (4) uterine enlargement^16^.

The selection criteria for this study were as follows: (1) adenomyosis-related symptoms, e.g., menorrhagia, dysmenorrhea, pelvic pain or bulk pressure; (2) an age younger than 45 years; (3) no clinical signs of the menopausal transition; (4) FSH and E_2_ levels typical of women of reproductive age; (5) no hormonal treatment within the 3 months preceding the study; (6) no history of malignancy; (7) no pelvic infection; (8) childbearing completed; (9) hysterectomy or other conservative treatments declined; and (10) normal ThinPrep cytology test results.

### Pre-ablation Preparations

Before ablation, all of the patients underwent examinations, including routine blood, serum gonadal hormone and CA125 testing.

In addition, uterine volume was recorded. The mean uterine diameter and volume were calculated using the following formulas: mean diameter = (length + width + height)/3 and volume = 4/3*π*r3, where r is the mean radius (mean diameter/2).

All of the patients were counseled on the potential risks and benefits of PMWA and on possible alternative treatments. All of the patients provided written informed consent.

### Instruments

A microwave tumor coagulator (KY-2000, Kangyou Medical Instruments Company, Nanjing, China) with a frequency of 2,450 MHz and continuous MW emission modes was used. The needle antenna had a 15-gauge external diameter (1.9 mm).

All of the hormone and CA125 assays were performed at a single (local) laboratory using the electrochemiluminescence method and standard laboratory techniques on a Cobas E601 analyzer (Roche, Germany).

### Therapy Procedures

**Figure 1:**
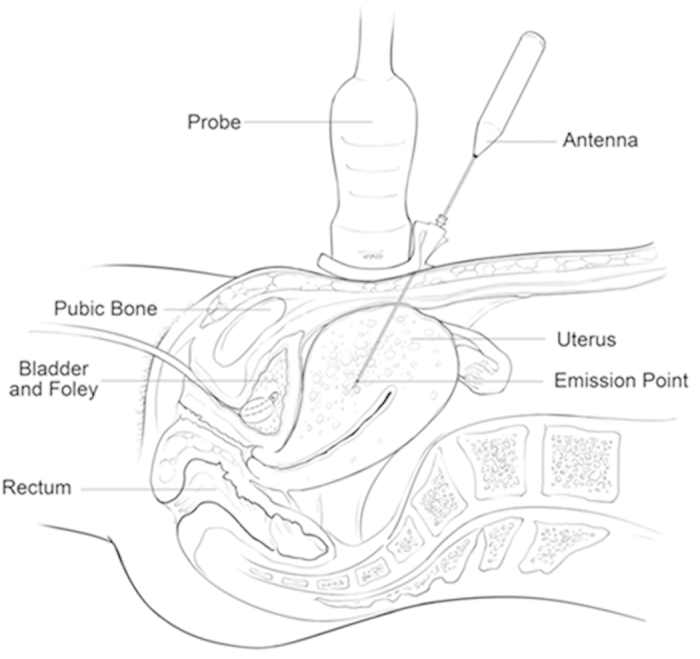
The diagram of PMWA.

**Figure 2:**
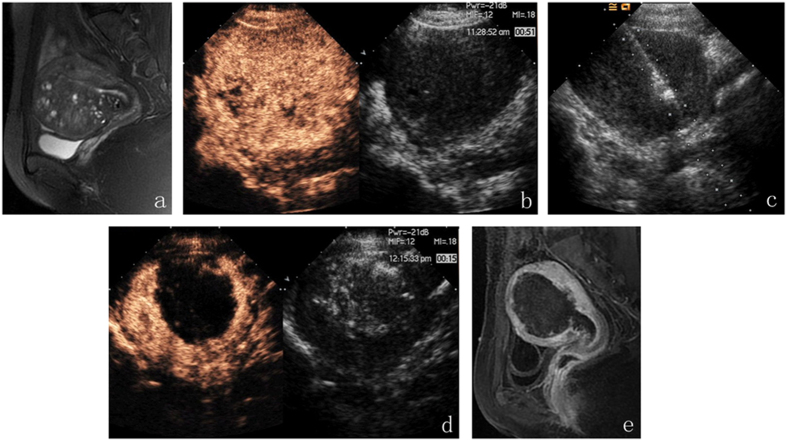
A 35-year-old woman with adenomyosis. Her uterus size was 9.3 cm*8.0 cm*8.7 cm. (**a**) MRI (T2WI): the enlargement of the junctional zone in the anterior wall. (**b**) CEUS before ablation: contrast agent perfused in the entire uterus. (**c**) The microwave energy radiated from the emission tip. (**d**) After the hyperechogenic signal covered at least 50% of the entire lesion, CEUS was performed to assess the volume of the non-perfused region. (**e**) Three days after PMWA, contrast-enhanced MRI was performed to evaluate both the efficacy of the ablation and the viability of the organs surrounding the uterus.

The patients adopted the supine position. Ablation was performed under intravenous conscious sedation (induction: midazolam 1.0 mg, fentanyl 0.05 ng, propofol 1.0–1.5 mg/kg; maintenance: propofol 0.4-1.2 mg/kg·h). Under the guidance of real-time US, the antenna needle was percutaneously inserted into the lesion (Fig. 1). Percutaneous approaches are associated with a risk of bowel puncture; therefore, the intestinal tract must be carefully pushed off from the abdominal wall using a probe before inserting the needle. Special attention should be paid to the shape of coagulation necrosis lesions, which are spheroid but not perfectly spherical. Therefore, the needle tip is not inserted into the center of the endometrial-myometrial area or adenomyoma but close to the periphery. Then, an output energy of 50 W or 60 W was selected for the ablation in most cases^5^. The entire procedure was performed under real-time US guidance. Once the hyperechogenic signal covered at least 50% of the entire lesion, the microwave energy was discontinued^5^. Then, the effectiveness of the ablation was immediately assessed using contrast-enhanced ultrasound (CEUS). The non-enhanced regions on contrast-enhanced sonography indicate necrotic areas^17^. When the non-enhanced area did not exceed 50% of the lesion, supplementary PMWA was immediately performed (Fig. 2).

### Clinical Follow-up

The patients were asked to return for re-evaluation on the same day of the menstrual cycle on which the ablation was performed (e.g., day 8, 9, 10, or 11). A total of 142 patients met these criteria. Measurements of uterine volume (based on transvaginal ultrasonography) and CA125, PRL, FSH and E_2_ levels were obtained 3, 6, 9 and 12 months after ablation. In addition, clinical signs of the menopausal transition and fertility-related data were evaluated.

### Statistical Analysis

All of the statistical analyses were performed using SPSS 13.0 (SPSS Inc., Chicago, IL, USA). Normality tests were applied for all of the continuous variables using the Shapiro-Wilk test. The Wilcoxon Matched-Pairs Signed-Rank Test was used to analyze the CA125 and serum hormone levels pre-ablation and at each follow-up visit.

A Spearman’s correlation coefficient analysis was used to determine the correlation between the CA125 and PRL levels and uterine volume.

Statistical significance was assumed at a *P*-value < 0.05.

## Results

The mean ( ± SD) age of the patients was 38.42 ( ± 4.07) years, and the ages ranged from 26–45 years. All of the patients were Chinese.

Regarding ovarian function after PMWA, the FSH and E_2_ results are shown in Table 1. No significant differences were detected between the levels pre-ablation and at follow-up. Because of losses to follow-up, the numbers of patients at 6, 9 and 12 months were 49, 33 and 39, respectively. One patient who was 44 years of age developed amenorrhea after ablation, but her FSH and E_2_ levels were in the normal range (E_2_ = 208 pmol/L and FSH = 7.9 U/L on the 3rd day of the menstrual cycle). Therefore, the abnormity in this patient was considered Asherman’s syndrome. The rest of the patients had regular, ovulatory menstrual cycles, and no menopausal transition symptoms were observed in this study.

Compared with the levels pre-ablation, a significant reduction in the CA125 levels was found during the follow-up period in patients with a uterine volume larger than 240 cm^3^ (Table 2). The median CA125 levels throughout the follow-up period exceeded 50 U/ml. The PRL levels declined in most of the patients: 137/142 patients (3 months), 46/49 patients (6 months), 31/33 patients (9 months) and 37/39 patients (12 months) (Table 1).

Based on the Spearman’s correlations, a significant correlation was found between the CA125 levels and a uterine volume larger than 240 cm^3^ (correlation coefficient = 0.486, *p* < 0.001). No correlation was found between the PRL levels and uterine volume or between the CA125 and PRL levels.

Seven patients gestated spontaneously after PMWA, and their fertility results are shown in Table 3.

## Discussion

In this study, the mean serum FSH and E_2_ values were not significantly different pre-ablation and during the follow-up period.

FSH is released from the pituitary gland to stimulate the growth of immature follicles^10^. A monotropic increase in FSH is the most consistently observed endocrine change in women who enter the perimenopausal period. This increase is the result of decreased negative feedback on the pituitary gland^18^. Low E_2_ levels under the influence of high FSH levels are indicative of diminished ovarian reserve^10^. PMWA is a method that uses in situ uterine ablation, which theoretically does not disturb blood perfusion in the ovaries. In this study, the mean serum FSH and E_2_ values were not significantly different pre-ablation and during the follow-up period. In addition, none of the patients complained of menopause-related clinical symptoms. Therefore, based on these observations in women younger than 45 years of age, PMWA does not have an observable effect on the ovaries within 1 year following the procedure. However, this study had a limitation. The levels of anti-Mullerian hormone (AMH), which is a more sensitive biomarker of ovarian reserve^19^,^20^,^21^, were not evaluated in this study; therefore, this protein warrants consideration in future studies.

In this study, the CA125 and PRL levels were correlated with adenomyosis and declined significantly following PMWA. CA125 mostly originates from ectopic endometriotic tissue and enters the systemic circulation^22^. A previous study demonstrated that high CA125 levels were consistently indicative of a severe disease status. This biomarker is important for monitoring the efficacy of conservative surgery for adenomyosis and endometriosis patients^2^,^12^. A study by Sheth and Ray on the relationship between adenomyosis and CA125 levels indicated that a larger uterine size was associated with a greater increase in CA125, especially in cases with particularly large uterine volumes (larger than 240 cm^3^)^12^. Therefore, in the present study, we assessed CA125 alterations in patients with a uterine volume larger than 240 cm^3^. The results revealed that the serum CA125 levels decreased significantly in patients with a uterine volume larger than 240 cm^3^. According to the Spearman’s correlation coefficient analysis, the downtrend was significantly correlated with a reduction in uterine volume, which was consistent with a previous study^12^. The CA125 values at each follow-up time point exceeded the standard cut-off value of 35 U/mL, which is used to detect epithelial ovarian cancer^23^. This finding may be due to diffuse adenomyotic lesions. PMWA causes local necrotic lesions; therefore, residual endometriosis may have been present after PMWA. It is difficult for serum CA125 levels to return to normal (non-endometriosis) levels.

PRL is a functionally versatile hormone with many unique effects and functions, including multiple reproductive and metabolic functions. Additionally, PRL is involved in tumorigenicity^14^, which may be due to its molecular heterogeneity^24^. Most of the research on PRL has been performed in rats. Mori *et al*. suggested that prolactin is a trigger in the genesis of adenomyosis^13^. Most research in humans has suggested that patients with endometriosis-related infertility are always hyperprolactinemic^24^, and the severity of endometriosis is associated with PRL secretion^25^. Our results did not indicate any correlations between PRL and CA125; however, the PRL levels decreased after PMWA in most of the patients.Furthermore, in contrast to our findings, treatment with danazol did not result in significant differences in basal PRL levels before and after therapy^26^. Therefore, further studies are needed to evaluate and answer these questions.

Patients who have a desire for children are not eligible for PMWA; however, most of the patients who undergo PMWA use birth control measures after treatment. In addition, women who present with adenomyosis are usually older than 35 years of age, and their fertility has begun to decline. Therefore, the results of this study suggest that patients remain fertile after PMWA. In the 7 patients with subsequent pregnancies, 4 chose artificial abortion, 1 is still pregnant, 1 had cervical pregnancy and 1 developed spontaneous abortion. Nevertheless, we did not define the last two conditions as complications from PMWA because these issues can occur in women with a healthy uterus. Therefore, it is difficult to estimate the gestation-specific and infertility risks of PMWA in this study. Synechia occurred at 1 patient in this study, less than 1%. Thus, PMWA might be an alternative treatment for women of reproductive age.

No interference with ovarian function was observed after US-guided PMWA. Both serum CA125 and PRL levels were significantly decreased after treatment. The correlation between uterine volume and CA125 levels was strong. While the relationship between the changes in PRL levels and PMWA warrant further study.

## In conclusion

US-guided PMWA is an effective method in the treatment of adenomyosis, has no significant impact on ovarian function and fertility and should be considered as an alternative to hysterectomy in women of reproductive age.

## Additional Information

**How to cite this article**: Yu, Y. *et al*. Ultrasound-guided percutaneous microwave ablation for adenomyosis: efficacy of treatment and effect on ovarian function. *Sci. Rep*. **5**, 10034; doi: 10.1038/srep10034 (2015).

## Figures and Tables

**Table 1:** 

	N	Pre-ablation M (P25, P75)	Post-ablation M (P25, P75)	*Z*	*p*
					
***E***_***2***_					
3 months	142	364.40 (212.94, 573.89)	397.63 (255.00, 533.30)	−0.084^a^	0.933
6 months	49	345.56 (217.93, 651.19)	350.00 (262.93, 600.92)	−0.016^a^	0.987
9 months	33	304.25 (204.72, 431.72)	400.88 (273.41,671.84)	−1.616^a^	0.106
12 months	39	326.32 (205.60, 573.01)	350.13 (244.22, 480.38)	−0.080^a^	0.936
					
***FSH***					
3 months	142	4.98 (3.44, 6.98)	4.89 (3.13, 6.49)	−0.594^a^	0.552
6 months	49	5.71 (3.70, 6.99)	5.14 (3.60, 6.68)	−1.048^b^	0.295
9 months	33	5.72 (3.73, 7.15)	4.59 (3.30, 6.84)	−0.817^b^	0.414
12 months	39	5.37 (3.65, 6.23)	5.59 (2.90, 8.24)	−0.305^b^	0.76
					
***PRL***					
3 months	142	21.35 (14.89, 32.27)	8.59 (6.65, 11.94)	−7.162^a^	< 0.001
6 months	49	23.69 (16.05,32.40)	8.00 (6.22, 11.65)	−4.914^a^	< 0.001
9 months	33	23.69 (13.35, 30.35)	10.01 (7.38, 13.04)	−3.736^b^	< 0.001
12 months	39	21.33 (13.55, 35.41)	9.53 (7.15, 15.38)	−3.582^a^	< 0.001

Serum hormone outcomes before and after PMWA.

M median; P:percentile.

*Z:* Z-score.

*p:* significance probability.

^a^Based on the positive ranks.

^b^Based on the negative ranks.

**Table 2:** 

	N	Pre-ablation M (P25, P75)	Post-ablation M (P25, P75)	*Z*	*p*
					
***CA125***					
3 months	56	107.45 (68.02, 210.78)	69.79 (48.52, 134.63)	−4.764^a^	< 0.001
6 months	29	108.30 (83.64, 184.90)	57.90 (39.66, 75.00)	−4.660^a^	< 0.001
9 months	20	125.80 (89.89, 210.78)	66.06 (48.43, 128.90)	−3.845^a^	< 0.001
12 months	17	146.90 (75.82, 243.95)	83.79 (58.93, 137.60)	−2.959^a^	0.003

Serum CA 125 before and after PMWA (uterine volume > 240 cm3).

M median.

*P*: percentile.

*Z:* Z-score.

*p:* significance probability.

^a^Based on the positive ranks.b Based on the negative ranks.

**Table 3:** 

Characteristics	Patients						
	1	2	3	4	5	6	7
Age (y)	29	36	38	39	43	39	39
Parturition	1	1	1		1	1	1
							
*Per-ablation*							
CA125(U/ml)	32.79	47.73	35.16	15.02	34.52	85.9	74.16
PRL	8.00	46.15	21.35	33.00	19.51	33.92	16.05
FSH	2.57	2.30	2.81	3.80	2.81	7.09	5.17
E_2_	233.51	336.28	754.78	410.93	469.71	1523.75	247.38
Uterine size (cm)	6.4*6.0*5.9	8.4*6.4*8.1	7.4*6.1*7.3	5.6*5.4*3.6	7.5*6.7*7.8	7.1*7.7*8.9	10.0*7.1*11.1
FAA (m)	16 and 20	10	10	12	13	21	14
gestation (w)	6 and 7	7		12	8	8	8
outcome	AA	CP	in pregnancy (26 weeks)	AA	SA	AA	AA

Spontaneous pregnancies after PMWA.

FAA: fertilization after ablation AA: artificialIinduced abortion.

SA: spontaneous abortion.

CP: cervical pregnancy.

m: month.

w: week.

## References

[b1] Garcia L. & Isaacson K. Adenomyosis: review of the literature. J. Minim. Invasive. Gynecol. 18, 428–437, 10.1016/j.jmig.2011.04.004 (2011). 21622029

[b2] Huang B. S. . Fertility outcome of infertile women with adenomyosis treated with the combination of a conservative microsurgical technique and GnRH agonist: long-term follow-up in a series of nine patients. Taiwan J. Obstet. Gynecol. 51, 212–216, 10.1016/j.tjog.2012.04.008 (2012). 22795096

[b3] Wood C. Surgical and medical treatment of adenomyosis. Hum. Reprod. Update. 4, 323–336 (1998). 9825848 10.1093/humupd/4.4.323

[b4] Rabinovici J. & Stewart E. A. New interventional techniques for adenomyosis. Best. Pract. Res. Clin. Obstet. Gynaecol. 20, 617–636, 10.1016/j.bpobgyn.2006.02.002 (2006). 16934530

[b5] Zhang J. . [Ultrasound-guided percutaneous microwave ablation in the treatment of diffuse adenomyosis]. Zhonghua yi. xue. za. zhi. 91, 2749–2752 (2011). 22322052

[b6] Zhang J. . Ultrasound-guided percutaneous microwave ablation for symptomatic uterine fibroid treatment--a clinical study. Int J Hyperthermia. 27, 510–516, 10.3109/02656736.2011.562872 (2011). 21756048

[b7] Yang Y. . Ultrasound-guided percutaneous microwave ablation for submucosal uterine fibroids. J. Minim. Invasive. Gynecol. 21, 436–441, 10.1016/j.jmig.2013.11.012 (2014). 24316137

[b8] Xia M. . Research of dose-effect relationship parameters of percutaneous microwave ablation for uterine leiomyomas--a quantitative study. Sci. Rep. 4, 6469, 10.1038/srep06469 (2014). 25267154 PMC4179463

[b9] Xia M. . Feasibility study on energy prediction of microwave ablation upon uterine adenomyosis and leiomyomas by MRI. Br. J. Radiol. 87, 20130770, 10.1259/bjr.20130770 (2014). 24947033 PMC4112404

[b10] Kaump G. R. & Spies J. B. The impact of uterine artery embolization on ovarian function. J. Vasc. Interv. Radiol. 24, 459–467, 10.1016/j.jvir.2012.12.002 (2013). 23384832

[b11] The American Society for Reproductive Medicine, Birmingham, Alabama. The menopausal transition. Fertil. Steril. 90, S61–65, 10.1016/j.fertnstert.2008.08.095 (2008). 19007648

[b12] Sheth S. S. & Ray S. S. Severe adenomyosis and CA125. J. Obstet. Gynaecol. 34, 79–81, 10.3109/01443615.2013.832178 (2014). 24359057

[b13] Mori T., Singtripop T. & Kawashima S. Animal model of uterine adenomyosis: is prolactin a potent inducer of adenomyosis in mice ? Am J Obstet Gynecol 165, 232–234 (1991). 1853904 10.1016/0002-9378(91)90258-s

[b14] Ben-Jonathan N., LaPensee C. R. & LaPensee E. W. What can we learn from rodents about prolactin in humans? Endocr. Rev. 29, 1–41, 10.1210/er.2007-0017 (2008). 18057139 PMC2244934

[b15] Hanafi M. Ultrasound diagnosis of adenomyosis, leiomyoma, or combined with histopathological correlation. J. Hum. Reprod. Sci. 6, 189–193, 10.4103/0974-1208.121421 (2013). 24347933 PMC3853875

[b16] Maheshwari A., Gurunath S., Fatima, F. & Bhattacharya, S. Adenomyosis and subfertility: a systematic review of prevalence, diagnosis, treatment and fertility outcomes. Hum. Reprod. Update. 18, 374–392, 10.1093/humupd/dms006 (2012). 22442261

[b17] Wang F. . Imaging manifestation of conventional and contrast-enhanced ultrasonography in percutaneous microwave ablation for the treatment of uterine fibroids. Eur. J. Radiol. 81, 2947–2952, 10.1016/j.ejrad.2011.12.037 (2012). 22341698

[b18] Klein N. A. . Age-related analysis of inhibin A, inhibin B, and activin a relative to the intercycle monotropic follicle-stimulating hormone rise in normal ovulatory women. J. Clin. Endocrinol Metab. 89, 2977–2981, 10.1210/jc.2003-031515 (2004). 15181087

[b19] van Rooij I. A. . Anti-mullerian hormone is a promising predictor for the occurrence of the menopausal transition. Menopause 11, 601–606 (2004). 15545787 10.1097/01.gme.0000123642.76105.6e

[b20] Tremellen K. P., Kolo M., Gilmore A. & Lekamge D. N. Anti-mullerian hormone as a marker of ovarian reserve. Aust. N Z J Obstet. Gynaecol. 45, 20–24, 10.1111/j.1479-828X.2005.00332.x (2005). 15730360

[b21] Fanchin R. . Serum anti-Mullerian hormone is more strongly related to ovarian follicular status than serum inhibin B, estradiol, FSH and LH on day 3. Hum Reprod 18, 323–327 (2003). 12571168 10.1093/humrep/deg042

[b22] Kitawaki J. . Usefulness and limits of CA-125 in diagnosis of endometriosis without associated ovarian endometriomas. Hum Reprod. 20, 1999–2003, 10.1093/humrep/deh890 (2005). 15890727

[b23] Bast R. C. Jr. . A radioimmunoassay using a monoclonal antibody to monitor the course of epithelial ovarian cancer. N Engl J Med. 309, 883–887, 10.1056/nejm198310133091503 (1983). 6310399

[b24] Wang H., Gorpudolo N. & Behr B. The role of prolactin- and endometriosis-associated infertility. Obstet Gynecol Surv. 64, 542–547, 10.1097/OGX.0b013e3181ab5479 (2009). 19624865

[b25] Machida T., Taga M. & Minaguchi H. Prolactin secretion in endometriotic patients. Eur J Obstet Gynecol Reprod Biol. 72, 89–92 (1997). 9076428 10.1016/s0301-2115(96)02649-8

[b26] Muse K., Wilson E. A. & Jawad M. J. Prolactin hyperstimulation in response to thyrotropin-releasing hormone in patients with endometriosis. Fertil Steril 38, 419–422 (1982). 6811338 10.1016/s0015-0282(16)46574-x

